# Whole-Foods, Plant-Based Diet Alleviates the Symptoms of Osteoarthritis

**DOI:** 10.1155/2015/708152

**Published:** 2015-02-28

**Authors:** Chelsea M. Clinton, Shanley O'Brien, Junwen Law, Colleen M. Renier, Mary R. Wendt

**Affiliations:** Department of Internal Medicine, Michigan State University College of Human Medicine, 15 Michigan Street NE, Grand Rapids, MI 49503, USA

## Abstract

*Objective*. To evaluate the effectiveness of a whole-foods, plant-based diet (WFPB) to reduce symptoms of osteoarthritis. *Methods*. Six-week, prospective randomized open-label study of patients aged 19–70 with osteoarthritis. Participants were randomized to a WFPB (intervention) or continuing current diet (control). Outcomes were assessed by mixed models analysis of participant self-assessed weekly SF-36v2 domain t scores, weekly Patient Global Impression of Change (PGIC) scales, and mean weekly Visual Analog Scale (VAS) pain assessment. Mixed models analysis also evaluated pre-post change from baseline level for standard clinical measures: weight, BMI, body temperature, pulse, and blood pressure. *Results*. Forty participants were randomized. Thirty-seven of them, 18 control and 19 intervention, completed the study. The intervention group reported a significantly greater improvement than the control group in SF-36v2 energy/vitality, physical functioning, role physical, and the physical component summary scale. The differences between the intervention and control PGIC scales were statistically significant over time. Intervention group improvement in VAS weekly mean was also significantly greater than that of the control group from week 2 onward. *Conclusion*. Study results suggest that a whole-foods, plant-based diet significantly improves self-assessed measures of functional status among osteoarthritis patients.

## 1. Introduction

Osteoarthritis (OA) is also known as degenerative arthritis or degenerative joint disease. It is a group of mechanical abnormalities involving the degradation of articular cartilage and subchondral bone in the joints. Symptoms primarily include pain, tenderness, and stiffness, though joint locking and effusions may also occur. A variety of causes including hereditary, developmental, metabolic, and mechanical etiologies may initiate the process of cartilage loss. As cartilage thins, bony surfaces become less well-protected and bone may be exposed or damaged. Regional muscles may experience atrophy and ligaments become more lax as a result of decreased movement secondary to pain. Treatment generally involves a combination of lifestyle modifications, exercise, and analgesics. Joint replacement may improve quality of life if pain becomes debilitating.

OA is the most common form of arthritis, affecting 27 million people in the United States. It is the third leading cause of years lived with a disability. 20% of adults in the United States report having doctor-diagnosed osteoarthritis [1].

The American Dietetic Association recognizes appropriately planned WFPB diets as healthful and nutritionally adequate. WFPB diets may provide benefits in the prevention and treatment of certain diseases [2]. WFPB dieters show increased levels of beta and alfa carotenes, lycopenes, lutein, vitamin C, and vitamin E in their sera [3]. An ordinary diet in the United States, represented by the USDA pyramid, includes solid and liquid animal protein ingested daily in the forms of meats and diary. Arachidonic acids are precursors to proinflammatory prostaglandins and leukotrienes. Western diets are high in arachidonic acids, derived primarily from consuming animal products, whereas WFPB diets are naturally low in arachidonic acids. A diet low in arachidonic acid levels has been shown to ameliorate the clinical signs of inflammation in patients with rheumatoid arthritis [4]. This kind of dietary habit is inexpensive, practical, and sustainable. A WFPB diet is easily understandable and requires limited educational materials. If proven effective to ameliorate the symptoms of osteoarthritis, this would be helpful to many people suffering with this disease.

Dietary treatment of rheumatoid arthritis is controversial. Several trials have assessed dietary modifications for rheumatoid arthritis. A number of studies have shown favorable benefit in rheumatoid arthritis symptoms with dietary restrictions of meat and dairy products [5–8]. One trial compared 12 months of a gluten-free WFPB diet consisting of vegetables, root vegetables, nuts, and fruits to an ordinary diet [9]. However, pain and functionality were not reported separately in patients with rheumatoid arthritis. Measurements of swollen joints, pain, and functional status were improved in the WFPB group compared to the nonvegan group after 3 months, 6 months, and 12 months. Two trials compared an elemental diet to an ordinary diet over four weeks. Neither study showed benefit in pain measurement or physical function [10, 11]. One randomized controlled trial compared a Cretan Mediterranean diet to an ordinary diet over 12 weeks, finding a significant improvement in pain, but no improvement in functionality or morning stiffness [12]. One trial of 53 patients compared an elimination diet to an ordinary diet over 6 weeks, but due to inadequate data reporting, no analyses were possible [13]. In another trial, patients were randomized to an allergen-free diet or an allergen-restricted diet. After four weeks, no difference between groups was found in morning stiffness [14]. Another trial including 17 patients compared an elemental diet to a well-mixed blended soup over three weeks, but no significant differences in pain or morning stiffness were found [15].

Two studies have specifically investigated dietary modification and fibromyalgia. One open, nonrandomized controlled study of 33 patients found beneficial effects of a vegan diet on fibromyalgia symptoms in a 6-week intervention period, with improvements in the Visual Analog Scale (VAS) and three other scales [16]. An observational study on a raw vegetarian diet with daily dehydrated barley grass juice supplementation showed benefits in 19 of 30 participants. Of those 19 responders, short-form health survey (SF-36) measures for all scales except bodily pain were no longer statistically different from normal women 45 to 54 years of age [9].

There have been no published prospective randomized control trials assessing whether a WFPB diet would benefit osteoarthritis.

*Purpose*. The specific aim of this study is to determine if a WFPB diet will result in a subjective reduction in pain and functional limitation in patients with osteoarthritis.

The following are hypothesized.

 The SF-36v2 BP, PF, RP, VT, and PCS improvement will be significantly greater in the intervention group than in the control group.
 Among participants capable of experiencing a clinically important level of change in VAS (>1.3), those with a day 1 VAS > 2, VAS improvement will be significantly greater in the intervention group than in the control group.
 PGIC scale improvement will be significantly greater in the intervention group than in the control group.

## 2. Methods

This study was a 6-week, prospective randomized open-label trial designed to assess the efficacy of WFPB diet, compared with ordinary diet, on the reduction of osteoarthritis symptoms. The clinical study site and protocol were approved by the Institutional Review Board. All patients provided written informed consent before the commencement of any study activities or procedures.

### 2.1. Subjects

Those eligible to enter the study were male and female community-dwelling patients previously diagnosed with osteoarthritis by a physician. People who were already on restricted diets at enrollment were excluded from the study.

Additional exclusionary criteria included ages less than 18 or greater than 70, history of an eating disorder, history of diabetes, inability to afford food, lack of control over the food eaten, pregnant or nursing, known food allergies, or patients following a medically prescribed diet. According to the 2010 Census of the United States, Grand Traverse County, MI was home to 86,986 people, 22.1% were under 18 years of age, and 10.4% were 70 years of age and over. Of the people in the county, after exclusion based on age, and estimating a 20% population frequency of arthritis, there were approximately 11,850 eligible for further study screening.

Seventy-six women and men, previously diagnosed with osteoarthritis by a physician, responded to newspaper, radio, website, and flyer advertising in Traverse City, Michigan. Of the 76 respondents, 40 were randomized and began the study; 37 completed the study, 19 in the intervention group and 18 in the control group. Of the 36 respondents who did not undergo randomization, 20 felt study participation was too difficult or were not interested after reviewing the informed consent, four were already on restricted diets, one was unable to make choices about the food s/he ate, seven were greater than 70 years of age, two had food allergies, and two had diabetes.

### 2.2. Design

Eligible recruits were emailed the informed consent and asked to contact the researchers if they were interested in study participation. Informed consent was signed by each participant at their intake interview. Using a four-square randomization tool, participants were assigned to either a WFPB diet or their ordinary omnivorous diet for a six-week period. The WFPB diet consisted of fruits, vegetables, legumes, and grains. Animal products were proscribed and the use of unrefined foods was encouraged. There was no restriction in energy intake in either diet group. Participants in the intervention group were encouraged to eat freely and not count calories. Participants were able to modify their medications according to their doctor's advice during the study period but were discouraged from undergoing significant modifications in their medical pain management program during the study. No meals were provided in the study. Compliance was monitored by weekly 24-hour food recalls by telephone.

The intervention group was trained on the WFPB diet at the intake interview for one hour in group lecture format and provided printed recipes and training materials from http://www.pcrm.org/. The intervention group was encouraged to obtain at least 90% of their calories from plants. The control group continued their normal omnivorous diet program. All participants were encouraged to contact researchers with any questions or concerns about their dietary modification by telephone or email and underwent additional individual consultations as needed to promote compliance and answer specific questions.

Current medications were noted at each office visit and during each weekly telephone call. Twenty-four hour food recall was obtained at intake, exit, and during weekly follow-up calls. Dietary intake of solid and liquid food products was also recorded. Adverse events were recorded during weekly telephone surveys and at exit interviews. Vital signs (sitting blood pressure, weight, height, pulse, and temperature) were obtained at the intake and exit interview. Compliance was measured by documentation of 24-hour food recall at intake and during weekly participant interviews. Participants notified the researcher of the number of meat and dairy servings consumed over the week at each weekly telephone contact and at intake and exit interviews.

### 2.3. Patient Reported Outcome (PRO) Measures

The response definitions used in this study were developed by the FDA in consultation with pharmaceutical companies and in accord with their recommendations for the development for OA therapies. The one-week version of the SF-36v2 Health Survey was used to measure 8 domains of health status: physical functioning (PF), role limitation due to physical problems, that is, role physical (RP), bodily pain (BP), general health (GH), energy/vitality (VT), social functioning (SF), and role limitations due to emotional problems, that is, role emotional (RE), and mental health (MH). Subscale for each domain was calculated following the standard SF-36v2 guidelines. *Z*-scores were computed for each of the domains based upon 2009 national norms. *T* scores were then computed for each domain subscale using the following equation: 50 + (*z*-score ∗ 10), thus resetting each domain distribution from a *z*-score distribution with a mean of zero and standard deviation of one to a *t* distribution with a mean of 50 and a standard deviation of 10. Aggregate Physical Component Summary Scale and Mental Component Summary Scale were created by computing the sum of all eight domain *z*-scores multiplied by published population weights. These aggregate values were then converted to PCS and MCS *t* scores using the equation: 50 + (agg_score ∗ 10). SF-36v2 data was collected at intake (baseline) and weekly for the following six weeks.

The Patient Global Impression of Change (PGIC) has two scales. The standard seven point scale (PGIC-Row) asks patients to rate their change in activity limitations, symptoms, emotions, and overall quality of life related to their OA since the start of the study, with 1 = “no change, or the condition has gotten worse” to 7 = “a great deal better, and a considerable improvement that has made all the difference.” The second scale (PGIC-Line) asks the patient to circle the number that matches their degree of change since starting the intervention on a number line, where 0 = “much better,” 5 = “no change,” and 10 = “much worse.” So, for PGIC-Row the score increases as things improve, and PGIC-Line decreases as things improve. Data for both PGIC-Row and PGIC-Line were collected during weekly telephone calls for the six weeks following study initiation.

A standard Visual Analog Scale (VAS) was completed by participants daily, from Day 1 to Day 42, at random times, rating their level of pain on a scale from 0 = “no pain” to 10 = “worst pain imaginable.”

## 3. Analysis

### 3.1. Baseline Assessment

Summary statistics were calculated for baseline data (Table 1). Quantitative data was expressed as the mean (SD), while nominal data is expressed as the number (percentage). The comparability between study groups for baseline quantitative data was evaluated using Student's *t*-test (two-tailed), while nominal data with binary classification was evaluated using Fisher's Exact Test (two-tailed), and nominal data with more than 2 categories was analyzed using the *χ* ^2^ test. Statistical significance was defined throughout the study as *P* < 0.05.

### 3.2. Primary Outcomes

**Figure 1:**
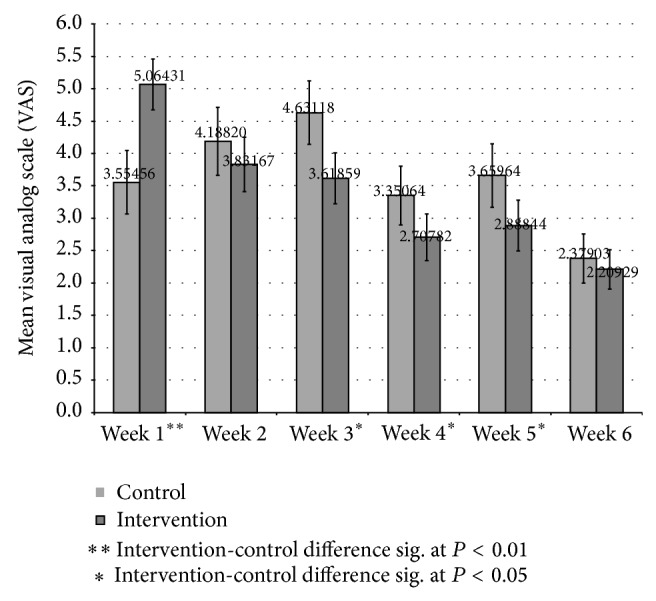
Mixed models (repeated measures) analysis of variance: mean weekly VAS pain participants with Day 1 VAS > 2 (9 control and 14 intervention).

The primary outcome variables for this study are changes in pain and functional status between entry into the study and the 6 weeks of enrollment in the study, as measured by the SF-36v2 domain subscales (*t*-scores) for bodily pain (BP), physical functioning (PF), role physical (RP), energy/vitality (VT) and Physical Component Summary (PCS) scores, VAS, and the PGIC scales. Mixed models (repeated measures) analysis was used to evaluate change in SF-36v2 subscales from intake (BL) and determine if the difference in change between the intervention and control groups was statistically significant (Table 2). Additional mixed models analysis of PGIC-Row and PGIC-Line data was conducted to evaluate description of life changes related to pain and rating of degree of change since BL. Because the PGIC measures change associated with participant's baseline pain and the VAS was not measured at intake, the participant's BL SF-36v2 BP subscale *t*-score was included as a covariate in analysis of PGIC-Row and PGIC-Line (Table 3). VAS data, which was only available for a subset of participants, was evaluated for participants with a VAS of 2 or greater on Day 1. Mixed models (repeated measures) analysis compared mean weekly VAS data (Figure 1). Mixed models analysis was also used to evaluate the change from Week 1 and determine if the weekly difference in change from week 1 between the intervention and control groups was statistically significant (Table 4).

### 3.3. Secondary Outcomes

Mixed models analysis was also used to evaluate change from baseline to exit interview for the intervention and control group clinical measures: weight, body-mass index (BMI), body temperature, pulse, and systolic and diastolic blood pressure, to determine if there was a significant intervention-control difference in level of change (Table 5).

## 4. Results

### 4.1. Baseline Demographics, Clinical Characteristics, and Surveys

The majority of study participants, 83.8%, were female, with 73% age 55 and over. No statistically significant demographic or clinical differences were identified at intake (baseline), but controls had a significantly higher mean SF-36v2 VT-Energy/Vitality subscale *t* score and reported slightly lower weekly meat and dairy consumption (Table 1).

### 4.2. Efficacy

The primary focus of the SF-36v2 analysis was the four domains and single component summary scale previously identified as directly related to pain and functional limitations: BP, PF, RP, VT, and PCS (Table 2). Intervention VT, PCS, and PF changes from baseline were greater than 10%, with the intervention-control differences statistically significant from weeks 1, 2, and 3, respectively. RP results were slightly less consistent, with the intervention-control difference statistically significant during weeks 2–4, and 6, but not week 5. BP demonstrated far less consistent findings, with significant group differences in change from BL only at weeks 2 and 4, but no end of study difference. The remaining domains not focused directly on functional limitations demonstrated either no significant group differences in change from BL or additional inconsistent findings. GH exhibited statistically significant intervention-control differences during weeks 3 and 6, while SF and RE only displayed differences in level of change during week 3 only. No statistically significant differences were identified in either MH or MCS.

Mean weekly PGIC-Row (description of change related to pain) and PGIC-Line (degree of change since beginning) scores, adjusted for SF-36v2 BP at BL, are shown in Table 3. No statistically significant intervention-control differences existed in either scale during week 1 of the study, with PGIC-Row means between 1.0 (no change) and 3.0 (a little better), and PGIC-Line means slightly better than 5.0 (no change) for both groups. By week 2, PGIC-Row intervention mean scores had increased by more than 50% to 4.0 (somewhat better), with the control group mean still hovering around 2.0 (almost the same), a statistically significant difference. By week 6, the control group mean still hovered around “almost the same,” while the intervention group mean had increased over 100% from week 1 to a level slightly in excess of 5.0 (moderately better). Control group PGIC-Line means changed very little over the 6 weeks of the study, while intervention group means decreased steadily over time, moving from 4.11 to 2.05 on a scale where 5 = “no change” and 0 = “much better.” The PGIC-Line intervention-control difference became statistically significant by week 3, continuing through week 6.

Mean Visual Analog Scale (VAS) pain assessments and associated 95% CI are presented by week in Figure 1. Participants were asked to provide a VAS assessment each day at a random time and a subset of participants, 13 controls and 15 intervention, provided the requested data. To be able to assess clinically important change in VAS, analysis was restricted to participants with a day 1 VAS of 2 or more, 9 controls and 14 intervention. Mixed models (repeated measure) analysis evaluating change in mean VAS assessments from Week 1 (Table 4) found that intervention group improvement was significantly greater than that of the control group for weeks 2 through 6. In addition, even though the intervention group mean was significantly higher than that of the control group during week 1, it was lower than the control group during weeks 2 through 6, with that difference being statistically significant during weeks 3 through 5.

Table 5 presents the changes in clinical findings from intake (BL) to exit interview. On average, the intervention group lost more weight than the control group, with associated lower body-mass index (BMI). No significant intervention-control differences were seen for change in body temperature, pulse, or blood pressure over the six-week intervention period.

## 5. Safety

Adverse events included one instance each of rash, herniated disc, dizziness, and a new diagnosis of prostate cancer in the control group. Within the treated group, adverse events included one urinary tract infection, 3 complaints of gas and bloating, one upper respiratory infection, one gouty arthritis exacerbation, and one complaint of hunger.

The incidence of adverse events in the intervention group was not higher than the control group.

## 6. Laboratory Findings

Initial CRP measurements were normal at intake screening laboratory evaluations in all participants. Subsequent readings after completion of the study did not differ significantly from initial measurements.

## 7. Discussion

WFPB diet was associated with a significant reduction in pain compared to an ordinary omnivorous diet, with statistically significant pain reduction seen as early as two weeks after initiation of dietary modification.

Previous studies show that diets enriched with omega-3 fats and plant proteins tend to decrease subjective complaints of pain in rheumatoid arthritis and fibromyalgia. Previous studies are limited by their design, size, significant dropout rates, and, subsequently, limited applicability. To our knowledge including exhaustive literature search, this is the first randomized, controlled trial to examine the effects of WFBP diet on subjective pain reports due to osteoarthritis.

The primary mechanism by which diet reduces subjective pain may be a result of normalization of the fatty acid profile and reduction in exposure to inflammatory protein precursors. Western diets are high in arachidonic acid, which are modified into proinflammatory prostaglandins and leukotrienes. Nonsteroidal anti-inflammatory drugs work to reduce pain by limiting the metabolism of arachidonic acid. Arachidonic acid is found in animal foods and some vegetable oils. Therefore, the adoption of a WFPB will dramatically reduce the availability of precursors necessary to produce painful prostaglandins.

In addition, WFPB dieters have higher serum levels of omega-3 fats than omnivores and even higher levels than fish eaters. The metabolism of alpha-linoleic acid, which is found in abundance in legumes, vegetables, and soy, produces anti-inflammatory prostaglandins. The decrease in concentration of these prostaglandins may contribute to reduction in symptoms in these patients.

Further, animal protein consumption results in the increased permeability in the small intestine, resulting in bacterial translocation that leads to immune complex development in the bloodstream. These bulky immune complexes can lodge in small capillaries, resulting in inflammation and damage accumulation over time. It is hypothesized that this is responsible for the exacerbation and proliferation of many autoimmune and chronic inflammatory diseases, such as arthritis conditions.

The plant-based dietary profile (low-fat, high fiber) can lead to a diet that is less energy dense and also results in a significant reduction in caloric intake. Despite reductions in calorie intake, the WFPB diet is associated with increased nutrient density as well as increased concentrations of several vitamins and trace minerals. Therefore, the WFPB diet group may have taken in fewer calories than the treated group while encouraged to eat to satiety without calorie counting. The reduction in mean body weight was achieved with no attempt to limit calorie intake.

The results are applicable outside the research setting because the participants were not provided food; they prepared their own meals or ate at restaurants. Further, study initiation techniques are easily duplicated in office settings as the intake interview training lecture and materials are available online.

The use of a WFPB diet offers several advantages that may facilitate compliance [17]. Because dairy, eggs, and meat are completely omitted, there is no need to measure portions, limit the size of meals, handle raw meat, or be concerned of raw meat safety precautions. A WFPB diet also appears to be easier to follow than previously studied raw diets and fasting, as evidenced by the high level of compliance and reasonable dropout rate of our study. Moreover, this diet elicited beneficial clinical results in as little as two weeks, which in turn will facilitate continued compliance.

The use of a plant-based diet is a source of concern for many people because of the common misconception that a diet without animal products will lead to malnutrition. Except for a very small risk of B12 deficiency, a WFPB diet based on unrefined plant foods supplies adequate amounts of calories, protein, fats, vitamins, and minerals including calcium, zinc, and iron [18].

The intervention group also experienced a statistically significant decrease in BMI and DBP. Weight loss and blood pressure reductions have been previously documented with WFPB diets [19, 20]. Weight loss could have contributed to the improvement in symptoms in the treatment group by decreasing the mechanical load on affected arthritic joints. For every pound of weight lost, there is a four-pound reduction in mechanical load exerted on the knee during daily activities [21]. Weight loss of 15 pounds has been shown to reduce knee pain by 50% in overweight individuals with arthritis [22]. However, most of the benefits of weight loss were associated with knee arthritis, and most of our subjects had more diffuse arthritis. Furthermore, half of the patients lost negligible amounts of weight (less than four pounds), while the other half of the treated patients lost between six and thirteen pounds. Weight loss was not consistent across the treatment group.

## 8. Study Limitations

A participant's level of discomfort from osteoarthritic disease may motivate dietary changes more than that felt by those with typical arthritic disease and asymptomatic patients. Study participants in nutrition research tend to be more knowledgeable about nutrition before study entry. However, the participants' level of support after the intake interview and lecture by telephone and email support was similar to that provided by other dietary and cooking interventions and considerably less than many previous studies. Some potential participants chose not to be in the study because it was too difficult, suggesting that people with less desire for desire for dietary change may have declined participation.

Sample size, study duration, and the size of the difference between treated and control groups limited the ability to show differences between the two groups. Statistically significant differences were identified within the intervention group over time participant adherence to instructed diet was based on self-report using a weekly 24-hour food recall. Actual patient adherence to dietary recommendations may be more or less than the reported adherence due to recall bias. We made extensive efforts to avoid expectation bias by giving the participants in the separate diet groups identical introductory and follow-up programs. The only thing that differed in the two groups was the dietary intervention and the one-hour lecture at the onset of the study.

The response to the intervention diet may have been blunted by the control group's knowledge about the dietary modification, which resulted in some members of the control group implementing some portions of the dietary modifications despite the researcher's request that they continue their original diet unmodified.

A placebo effect may also have affected the control group since they received increased attention during the study period by getting one hour of additional instruction at the start of the study. They were encouraged to share questions or concerns each week at their follow-up calls, but this effect may have been equally significant in the intervention group, since they were also encouraged to share questions or concerns. The intervention group received the same amount of telephone calls and office visits during the study period but received only the additional one-hour lecture and paper handouts at the onset of the study. The beneficial effects of WFPB diets on mood and depression are reported in previous studies [23, 24]. These psychological effects may modify responses to pain. However, the SF-36 did not identify changes in emotional health, suggesting psychological complications were not realized in the current study.

The short study period may be a limitation, but it does not diminish the importance of the findings. The biggest limitation is that the study was not a crossover design, which would have allowed both groups to receive treatment, but in different order. Several previous nutrition studies have shown support for short-term interventions, suggesting that nutrition interventions do not require a long duration to show benefit. For patients with active chronic kidney disease, a vegetarian diet with reliance on grains as the primary protein source resulted in decreased serum phosphate concentrations in one week [25]. When a woman begins a low-fat diet, serum estrogen concentrations decrease by 15–50% in 2-3 weeks [26, 27]. Dietary modification has resulted in increased functional capacity, decreased systolic and diastolic blood pressure, and decreased cholesterol in twelve weeks [19, 20]. Of patients with mild, functionally limiting angina, 74% were pain-free after twelve weeks of dietary change [28]. Significant modulation of biological processes that have critical roles in tumorigenesis is seen three months after dietary change [29].

The short study period does limit our ability to show differences between the intervention and treated groups. More study participants and a longer duration of study may identify a greater difference in improvement over time and between groups. A longer study period may have resulted in the accumulation of more data points, so that further comparisons between groups would have been possible, especially after the application of RAMCOVA.

The shorter duration of the study, limited number of study participants, and relatively small difference in improvement over the study limited the ability of the study to show change between intervention and control groups over time. Statistically significant improvements were shown within the intervention group over time. Statistically significant differences between intervention and control may have been possible if the study was larger or of longer duration.

## 9. Conclusion

The present and earlier studies provide further evidence for the beneficial effects of WFPB diets in many patients with OA. We hope that the results from the current study will encourage an increased appreciation and clinical evaluation of dietary variables and that WFPB diet therapies are recommended as an adjunct to standard medical management of this debilitating chronic disease.

## Figures and Tables

**Table 1:** Baseline assessments.

Description	Control	Intervention	*P* value
	(*n* = 18)	(*n* = 19)	
Demographic			
Sex: female^‡^: # (%)	16 (88.9)	15 (78.9)	0.66
Age^†^: mean (Std. dev.)	60.0 (6.3)	56.1 (8.4)	0.12
Race: white^‡^: # (%)	18 (100.0)	16 (88.9)	0.49
Smoker: yes^‡^: # (%)	1 (5.6)	0 (0.0)	1.00
Health conditions^‡^: # (%)			
Hypertension: yes	5 (27.8)	6 (33.3)	1.00
Depression: yes	8 (44.4)	5 (27.8)	0.49
Clinical exam findings^†^: mean (Std. dev.)			
BMI	28.4 (4.5)	29.1 (6.5)	0.72
Body temperature	98.4 (0.9)	98.2 (0.7)	0.68
Pulse	75.7 (6.8)	72.6 (9.4)	0.27
Blood pressure: systolic	127.6 (17.6)	123.8 (16.6)	0.51
Blood pressure: diastolic	76.1 (7.6)	74.1 (10.2)	0.49
Survey responses			
Meat and dairy per week^§^: # (%)			
5–8/week	5 (27.1)	0 (0.0)	**0.049**
9–12/week	2 (11.1)	4 (22.2)	
>12/week	11 (61.1)	14 (77.8)	
SF-36v2 *t* scores^†^: mean (Std. dev.)			
PF: physical functioning	44.2 (10.5)	39.7 (10.6)	0.21
P: role physical	45.8 (9.9)	40.8 (10.4)	0.15
BP: bodily pain	40.1 (8.9)	39.2 (7.0)	0.72
GH: general health	49.5 (10.3)	44.4 (10.4)	0.15
VT: energy/vitality	47.6 (9.9)	40.8 (7.9)	**0.03**
SF: social functioning	43.3 (10.6)	41.7 (8.6)	0.62
RE: role emotional	44.7 (10.9)	43.9 (9.5)	0.81
MH: mental health	44.2 (11.4)	42.4 (11.5)	0.64
PCS: Physical Component Summary Scale	45.0 (8.5)	40.2 (10.5)	0.13
MCS: Mental Component Summary Scale	45.4 (11.2)	43.9 (10.8)	0.69

^‡^Fisher's exact test (two-tailed).

^†^Student's *t*-test (two-tailed).

^§^Chi-square test of significance.

**Table 2:** SF-36v2^*^ mixed models analysis of change from baseline.

	PF	RP	BP	VT	PCS	GH	SF	RE	MH	MCS
Baseline mean										
Control (Cntl)	44.15	45.75	40.13	47.63	45.04	49.48	43.26	44.74	44.19	45.36
Intervention (Int)	39.66	40.8	39.18	40.77	40.19	44.42	41.69	43.92	42.44	43.93
Change from baseline										
Week 1										
Cntl	0.33	2.23	2.23	−0.06	1.78	1.20	4.03	2.03	0.12	0.84
Int	4.97	1.80	2.59	5.88	3.21	3.46	7.37	1.64	5.35	4.79
Int-Cntl	4.64^‡^	−0.43	0.36	5.94^†^	1.43	2.26	3.33	−0.40	5.24	3.95
Week 2										
Cntl	0.88	1.49	2.11	3.89	−0.23	1.17	6.88	4.56	5.72	6.97
Int	4.20	5.40	5.77	9.29	4.57	4.23	7.64	3.59	6.85	6.87
Int-Cntl	3.31	3.91^†^	3.66^†^	5.41^†^	4.80^‡^	3.06	0.76	−0.98	1.13	−0.11
Week 3										
Cntl	0.19	−1.11	2.89	1.29	−0.19	−1.61	2.61	−0.63	2.87	1.77
Int	6.68	7.91	5.50	10.08	5.85	4.57	9.06	6.58	7.29	8.09
Int-Cntl	6.49^‡^	9.02^¥^	2.62	8.79^‡^	6.04^‡^	6.19^†^	6.45^†^	7.20^‡^	4.42	6.32^†^
Week 4										
Cntl	−0.14	1.96	1.92	2.94	−0.35	1.14	6.76	2.17	6.03	5.95
Int	7.54	8.24	8.15	11.08	6.63	4.53	9.96	6.99	10.14	9.67
Int-Cntl	7.68^¥^	6.28^‡^	6.24^†^	8.14^†^	6.98^¥^	3.39	3.20	4.82	4.11	3.72
Week 5										
Cntl	1.89	4.12	6.25	4.69	2.28	1.06	6.49	5.29	5.99	6.46
Int	6.14	7.91	7.63	12.44	5.99	5.02	10.19	7.57	8.60	9.93
Int-Cntl	4.24^†^	3.79	1.38	7.76^†^	3.71^†^	3.96	3.69	2.28	2.61	3.47
Week 6										
Cntl	1.02	2.65	5.41	5.49	1.31	2.01	5.08	5.05	6.46	6.87
Int	7.11	9.29	8.61	11.97	7.44	7.15	10.47	7.97	9.48	9.97
Int-Cntl	6.09^‡^	6.64^‡^	3.20	6.49^†^	6.13^‡^	5.14^†^	5.39	2.92	3.01	3.10

^*^ *t* scores based upon US National Norms: mean = 50 and SD = 10.

^†^ *P* < 0.05; ^‡^ *P* < 0.01; ^*¥*^ *P* < 0.001.

**Table 3:** Mixed models analysis of PGIC change from Week 1^‡^.

	Control	Intervention	Int-Cont	*P* value
PGIC-Row				
Week 1	1.72	2.63	0.91	0.073
Change from Week 1				
Week 2	0.33	1.37	1.04	0.022
Week 3	0.00	1.79	1.79	0.002
Week 4	0.00	2.21	2.21	<0.001
Week 5	0.28	2.32	2.04	0.003
Week 6	0.54	2.74	2.19	0.001
PGIC-Line				
Week 1	4.67	4.11	−0.56	0.127
Change from Week 1				
Week 2	−0.06	−0.26	−0.21	0.541
Week 3	0.67	−0.63	−1.30	0.016
Week 4	0.11	−1.42	−1.53	0.008
Week 5	−0.17	−1.37	−1.20	0.018
Week 6	−0.01	−2.05	−2.04	<0.001

^‡^Adjusted for BL SF-36v2 BP.

**Table 4:** VAS mixed models (repeated measures) analysis of change from Week 1 participants with Day 1 VAS >2.

	Control	Intervention	Intervention-control	*P* value
	(*n* = 9)	(*n* = 14)		
Week 1	3.56	5.06	1.51	<**0.001**
Change from Week 1				
Week 2	0.63	−1.23	−1.87	<**0.001**
Week 3	1.08	−1.45	−2.52	<**0.001**
Week 4	−0.20	−2.36	−2.15	<**0.001**
Week 5	0.10	−2.18	−2.28	<**0.001**
Week 6	−1.18	−2.85	−1.68	<**0.001**

**Table 5:** Clinical findings: mixed models analysis of change from baseline.

	Intake (BL)		Exit: change from BL			*P* value
	Control (Cntl)	Intervention (Int)	Cntl	Int	Int-Cont	
Weight (lbs.)	167.02	180.48	0.89	−5.23	−6.13	**0.003**
BMI (kg/m^2^)	28.41	29.07	0.17	−0.84	−1.01	**0.006**
Body temperature (F°)	98.36	98.24	−0.01	−0.02	−0.02	0.957
Pulse	75.67	72.76	0.78	0.93	0.16	0.961
Systolic BP (mmHg)	127.61	123.84	−2.50	−9.37	−6.87	0.124
Diastolic BP (mmHg)	76.11	74.05	−1.94	−4.21	−2.27	0.426
